# Clinical formulas, mother's opinion and ultrasound in predicting birth weight

**DOI:** 10.1590/S1516-31802008000300002

**Published:** 2008-05-01

**Authors:** Maria Regina Torloni, Nelson Sass, Jussara Leiko Sato, Ana Carolina Pinheiro Renzi, Maísa Fukuyama, Paula Rubia de Lucca

**Keywords:** Fetal weight, Ultrasonography, prenatal, Birth weight, Uterus, Organ size, Peso fetal, Ultra-sonografia pré-natal, Peso ao nascer, Útero, Tamanho do órgão

## Abstract

**CONTEXT AND OBJECTIVE:**

Accurate fetal weight estimation is important for labor and delivery management. So far, there has not been any conclusive evidence to indicate that any technique for fetal weight estimation is superior to any other. Clinical formulas for fetal weight estimation are easy to use but have not been extensively studied in the literature. This study aimed to evaluate the accuracy of clinical formulas for fetal weight estimation compared to maternal and ultrasound estimates.

**DESIGN AND SETTING:**

Prospective study involving 100 full-term, cephalic, singleton pregnancies delivered within three days of fetal weight estimation. The setting was a tertiary public teaching hospital in São Paulo, Brazil.

**METHODS:**

Upon admission, the mother's opinion about fetal weight was recorded. Symphyseal-fundal height and abdominal girth were measured and two formulas were used to calculate fetal weight. An ultrasound scan was then performed by a specialist to estimate fetal weight. The four estimates were compared with the birth weight. The accuracy of the estimates was assessed by calculating the percentage that was within 10% of actual birth weight for each method. The chi-squared test was used for comparisons and p < 0.05 was considered significant.

**RESULTS:**

The birth weight was correctly estimated (± 10%) in 59%, 57%, 61%, and 65% of the cases using the mother's estimate, two clinical formulas, and ultrasound estimate, respectively. The accuracy of the four methods did not differ significantly.

**CONCLUSION:**

Clinical formulas for fetal weight prediction are as accurate as maternal and ultrasound estimates.

## RESUMO

**CONTEXTO E OBJETIVO:**

A avaliação correta do peso fetal é importante na assistência ao trabalho de parto. Até o presente, não existe evidência conclusiva que aponte que algum método de estimar o peso fetal seja superior aos outros. As fórmulas clínicas usadas para estimar o peso fetal são de fácil realização, porém não têm sido extensivamente estudadas na literatura. Este estudo visou avaliar a acurácia das fórmulas clínicas na predição do peso fetal, comparadas ao peso estimado através da opinião materna e da ultra-sonografia e ao peso ao nascer.

**TIPO DE ESTUDO E LOCAL:**

Estudo prospectivo envolvendo 100 gestantes de termo, com feto único e cefálico, que tiveram seus partos dentro de três dias da estimativa do peso fetal. O estudo foi realizado em maternidade ensino, pública e terciária, na cidade de São Paulo, Brasil.

**MÉTODOS:**

Ao ser internada, a opinião da gestante acerca do peso fetal foi registrada. Mediu-se a altura uterina e a circunferência abdominal maternas e esses números foram usados em duas fórmulas clínicas para se estimar o peso fetal. Um especialista realizou então uma ultra-sonografia para estimativa do peso fetal. As quatro estimativas foram comparadas com o peso ao nascer e a acurácia de cada método foi avaliada comparando-se a porcentagem de estimativas dentro de 10% do peso ao nascimento. O teste do χ^2^ foi usado para comparações e p < 0,05 considerado significante.

**RESULTADOS:**

O peso ao nascer foi corretamente estimado (± 10%) em 59%, 57%, 61% e 65% das vezes através da opinião materna, das duas fórmulas clínicas e da ultra-sonografia, respectivamente. Não houve diferença significante na acurácia dos quatro métodos.

**CONCLUSÕES:**

As fórmulas clínicas são tão precisas na avaliação do peso fetal quanto a opinião materna e a ultra-sonografia.

## INTRODUCTION

Correct estimation of fetal weight, along with gestational age and the adequacy of the mother's pelvis, is important information for managing labor and delivery. According to the existing literature, there is no truly accurate technique for evaluating fetal weight. Until the early 1980s, fetal weight estimation (FWE) relied exclusively on clinical methods based on abdominal palpation and uterine measurements. Since the advent of ultrasound and its dissemination over the last three decades, and despite the lack of conclusive evidence, there has been a widespread belief that ultrasound is more accurate than other methods for predicting fetal weight. However, since 1990, several papers have reported that weight estimates using abdominal palpation and even the mother's opinion were as accurate as ultrasound FWE,^1–4^ with the advantage of being inexpensive and available at any time.

The estimation of fetal weight through abdominal palpation (using Leopold's maneuvers) is subjective and is therefore somewhat difficult to teach, especially to younger physicians and midwives. Clinical methods for FWE using fundal height^5^ and maternal abdominal girth measurements^6^ are objective and easy to teach. However, these clinical methods for FWE have not been extensively studied and there are few papers evaluating the accuracy of FWE derived from abdominal measurements compared with ultrasound or maternal estimates.^7,8^

The development and validation of simple, effective and inexpensive tools for reproductive health are important worldwide and especially relevant in developing countries, where high-cost equipment and trained technicians are scarce.

## OBJECTIVE

The objective of this study was to evaluate the accuracy of two clinical formulas used for fetal weight estimation, compared with maternal and ultrasound estimates and with the weight at birth, in full-term pregnancies.

## METHODS

This was an accuracy study conducted at a large tertiary public teaching maternity hospital that serves low-income women in the city of São Paulo, Brazil. The study was approved by the Institution's Ethics Committee and was conducted between July and October 2005.

All pregnant women admitted to the labor ward at full term (≥ 37 weeks), with a live singleton fetus in cephalic presentation and intact membranes were eligible. Patients in the first stage of spontaneous labor, as well as those admitted for elective induction or cesarean section were included. The exclusion criteria were multiple gestations, non-cephalic presentations, oligohydramnios or polyhydramnios, uterine fibroids and known fetal malformations. Patients were not excluded due to maternal conditions such as hypertensive disorders, diabetes or obesity. The patients were not consecutive since all clinical FWE were done personally by four specific second-year obstetrics-gynecology residents, during their shifts in the labor ward.

After admission, all eligible patients were contacted by one of the four residents and invited to participate in the study. After giving written informed consent, each woman was asked, “How much do you think your baby weighs?” and this information was recorded on a data sheet kept separate from the patient's chart.

The patient was then asked to empty her bladder and the resident measured her symphyseal-fundal height (SFH) and abdominal girth (AG), between contractions, using a flexible, non-elastic, standard sewing tape. Both measurements were performed with the patient lying flat on her back, with her legs extended, and were rounded to the nearest centimeter. The fundal height was measured from the midpoint of the upper border of the pubic symphysis to the highest point of the uterine fundus. The upper hand was placed firmly against the top of the fundus with the measuring tape passing between the index and middle fingers and readings were taken from the perpendicular intersection of the tape with the fingers.^5^ For the AG measurement, the tape was repositioned to encircle the woman's waist, at the level of the umbilicus, without applying excessive pressure to tighten the tape around the abdomen.^6^

The resident then performed a pelvic examination to evaluate cervical dilation and the degree of descent of the fetal head into the pelvis. The fetus was considered to be at a minus station when the lowermost portion of the fetal head was above the ischial spines, at zero station (engaged) when the vertex was at the level of the spines and at a plus station when it was below this level. Both measurements (SFH and AG) and information on the fetal station were recorded on the individual data sheet and later used to calculate the fetal weight according to the formulas proposed by Johnson and Toshach^5^ and Dare et al.^6^ (Chart 1).

**Chart 1.** Clinical formulas for fetal weight estimation.

**Johnson's formula:** Fetal Weight in grams* = 155 x (Fundal height in cm† – K) K = 11 (fetal head at plus stations) K = 12 (fetal head at zero station) K = 13 (fetal head at minus stations)
**Dare's formula:** Fetal weight in grams = fundal height in cm x abdominal girth in cm

* Johnson's original formula converted to grams, where ounces were multiplied by 28.34 and pounds were multiplied by 0.453;

† for patients over 90 kg, subtract 1 from the fundal height.

The patient was then sent to the Radiology department, for an obstetric ultrasound scan that included electronic caliper measurement of the fetal head, abdomen and femur, as well as amniotic fluid and placental evaluation. All ultrasound scans were performed by one of three experienced sonologists who were members of the hospital staff. The ultrasound fetal weight was calculated automatically by the equipment, using Hadlock's reference table, which used the biparietal diameter, abdominal circumference and femur length.^9^ All examinations were performed on the Logic Pro 400 ultrasound equipment (General Electric, United States), using a convex 3.5 MHz transducer. A printed report on the ultrasound examination, including all measurements and FWE, was attached to the patient's chart. The sonologist did not have access to the patient's data sheet containing the mother's estimate and clinical measurements. The residents and patients were instructed not to discuss their estimates with the sonologist.

All participants delivered within the next three days following the FWE, and the infants were weighed using a digital balance, immediately after birth, by the attending nurse, who recorded the information on the infant's and the mother's charts. The infants’ actual birth weight, the ultrasound estimate and relevant maternal data (admission history, ethnicity, age, parity, weight and height) were retrospectively retrieved by the authors from the patients’ charts, after discharge.

Gestational age was based on the time when the last reliable menstrual period occurred, ultrasound performed before reaching 20 weeks or neonatal examination. The mother's body mass index (BMI) was calculated by dividing her weight at admission (kg) by her height squared (m^2^). This “late pregnancy” BMI was used (instead of pre-pregnancy or first trimester BMI) in order to determine the effect of the current BMI on the FWE.

The accuracy of the four methods for FWE was assessed by calculating the percentage of the estimates that were within 10% of the actual birth weight for each method. Assuming that the ultrasound estimate of fetal weight would be accurate (within 10% of the actual birth weight) for at least 60% of the time,^4^ 90 subjects would be needed to show a difference in accuracy of at least 15% by means of clinical or maternal estimations, with 80% power and α of 0.05. Chi-squared analysis was used to determine whether the percentage of estimates within 10% of the actual birth weight was different between the clinical, maternal and ultrasound estimates of birth weight. A p-value < 0.05 was considered significant.

## RESULTS

A total of 132 full-term patients were approached by the authors during the study period. Eleven refused to participate and 21 did not enter the study for various reasons (second stage of labor, breech presentation, ruptured membranes, fetal demise, fetal malformation, abnormal amniotic fluid volume or delivery more than three days after admission), thus leaving 100 participants. During the same period, 1,239 patients were delivered at the maternity hospital.

Table 1 presents maternal and infant demographics. 42% of the women were nulliparae. Most women (70%) were Caucasian, 24% were obese (> 90 kg) and 77% had received prenatal care at local public outpatient clinics. Hypertensive disorders were present in 30 patients, five were diabetics and 15 had various other maternal complications (anemia, epilepsy, thyroid disease, cardiac disease, lupus or deep vein thrombosis). Most participants (63%) were in spontaneous labor, while 37% were admitted for elective induction or cesarean section. The mean fundal height (± standard deviation, SD) was 35.7 ± 2.8 cm (range 28-43) and the mean maternal abdominal circumference was 102.6 ± 10.3 cm (range 83-130). There were 13 neonates that weighed over 4,000 g.

**Table 1.** Maternal and infant demographics of the 100 pregnancies that underwent fetal weight estimation

Characteristics	Mean ± SD	Range
Age (y)	26.7 ± 7.6	14-45
Parity	1.2 ± 1.5	0-6
Maternal weight (kg)	78.2 ± 18.2	53.3-130.0
Maternal height (m)	1.60 ± 0.07	1.30-1.77
Admission BMI	30.5 ± 5.8	19.7-40.0
Gestational age (weeks)	39.2 ± 1.1	37.0-41.6
Birth weight (g)	3361 ± 541	2050-4880
Dare's estimate (g)	3678 ± 575	2324 -5074
Johnson's estimate (g)	3520 ± 436	2325-4495
Maternal estimate (g)	3158 ± 454	2000-4300
Ultrasound estimate (g)	3293 ± 545	2030-4545

SD = standard deviation; BMI = body mass index; referring to maternal weight at admission (kg)/maternal height squared (m)^2^.

The accuracy of the four methods for predicting the actual infant birth weight is presented in Table 2. No significant differences in the percentage of estimates within 10% among the four methods were detected using chi-squared analysis (p > 0.05).

**Table 2.** Clinical, maternal and ultrasound estimates of fetal weight in 100 patients

Method weight	Mean absolute simple error	Mean standardized absolute error	Estimates within 10% of birth
	± SD (g)	± SD (%)	(%)
Dare's formula	436 ± 359	14 ± 12	57
Johnson's formula	335 ± 234	11 ± 8	61
Mother's opinion	355 ± 320	11 ± 9	59
Ultrasound	312 ± 229	9 ± 7	65

Absolute simple error = estimate - actual birth weight. Standardized absolute error = (value of absolute simple error/actual birth weight) x 100. SD = standard deviation.

The percentage of correct estimates by nulliparous women did not differ significantly from that of parous women (57% versus 68%, p = 0.38).

## DISCUSSION

Fetal weight estimation using a measuring tape and two different clinical formulas was as accurate as maternal or ultrasound estimates for predicting the infant's actual birth weight within 10%.

In their original 1954 publication, Johnson and Toshach^5^ reported that fetal weight was within 353 g (12.45 oz) of the actual birth weight in 68% of their 200 cases. In the present study, using the same formula, 57% of the estimates were within this range. One possible explanation for this difference may be that maternal obesity (> 90 kg) was much more frequent in the present study than in Johnson and Toshach's work (24% versus 5.5%). It must be pointed out that the original inventors of the formula derived their correction factor for obese women (1 cm) using only 11 cases. It is possible that maternal adiposity may have a greater impact on FWE than originally thought and perhaps the proposed correction factor should be reevaluated in a larger sample of obese women. Indeed, Sauceda González et al.^10^ reported in a multicenter study involving 504 full-term patients that, while the overall mean fetal weight estimated through Johnson's formula did not differ significantly from the actual birth weight, the mean estimated fetal weight among obese women (admission BMI > 29.9) was significantly different from the mean actual birth weight (p = 0.0002).

In 1990, Dare et al.^6^ proposed a simpler formula for clinical FWE, which consisted of multiplying SFH by AG. In their original paper, Dare et al. tested this method on 498 full-term patients and obtained a good correlation between the clinical estimate and actual birth weight (r = 0.742). In the present study, Dare's formula was slightly less accurate than Johnson's formula. This may be explained by the lack of correction for obesity in Dare's model and the high prevalence of women > 90 kg in our study population. Larger studies involving obese patients are needed to test the hypothesis that Dare's formula for FWE is less accurate in these women.

Using Dare's formula and Johnson's formula, the residents involved in the present study correctly predicted birth weight (± 10%) in 57% and 61% of the cases, respectively. These values are very similar to the 54%-70% rate of correct estimates (± 10%) reported from studies that used abdominal palpation for FWE.^11^ There are no large studies comparing the accuracy of FWE estimation using clinical formulas versus abdominal palpation. The decision to use clinical formulas instead of Leopold's maneuvers was due to concerns about the relative inexperience of the residents and the paucity of published studies on the validation of these clinical methods for FWE. In practice, both Dare's method and Johnson's method were easy to perform and teach. Previous papers have indicated that the inter and intraobserver variability of uterine height measurements is small, ranging from 0.52 cm to 1.72 cm.^12^ In the largest study evaluating the accuracy of FWE through clinical palpation (n = 661 full-term patients), Chauhan et al.^13^ reported a mean absolute weight error of 367 g and a mean absolute percentage error of 10.3, which are very similar to the numbers obtained using Johnson's formula in the present study. Assuming that the accuracy of FWE using Leopold's maneuvers and Johnson's method is similar, it seems that since Johnson's method relies on objective measurements and calculations, this latter method of FWE may be easier to perform and teach, especially to midwives or less experienced examiners such as medical students or residents.

While other studies have confirmed that Johnson's formula correctly predicts actual birth weight^14–17^ only two previous papers have compared the accuracy of this formula with ultrasound estimates. In a study involving 46 patients, Banerjee et al.^8^ did not find significant differences in the mean absolute simple error and mean standardized error of FWE using Johnson's formula or ultrasound. Similarly, Cury and Garcia^7^ reported that FWE using Johnson's formula was as accurate as ultrasound estimates. There are no published studies comparing the accuracy of Dare's formula versus ultrasound FWE.

The percent of ultrasound predictions within 10% of the actual birth weight obtained in the present study (65%) is within the reported range (23-78%).^18^ While some studies have indicated that ultrasound FWE is superior^19,20^ or inferior^2,21^ to clinical predictions, most have reported that the two methods have similar accuracy.^1,4,8,17,22^ In the largest study comparing ultrasound versus clinical FWE, Chauhan et al.^13^ did not find significant differences in 460 patients at gestational ages of between 37 and 40 weeks. While clinical estimates were correct (± 10%) in 61.7% of their cases, ultrasound estimates were correct in 60%. Their values were very similar to the present findings.

In developing countries, it is important to remember that ultrasound FWE requires expensive equipment and is time-consuming for the hospital staff performing the examinations, who are often working in suboptimal conditions and overcrowded maternity facilities. Requesting costly ultrasound estimates is hardly justifiable when clinical and maternal estimates are equally accurate and can be quickly carried out at no cost.

Since Chauhan et al.^1^ first published their study in 1992, several studies have confirmed that maternal estimates are as accurate as ultrasound estimates.^3,4,11^ The present study is the first to evaluate maternal FWE among a Brazilian low-income population. The accuracy of maternal estimates ± 10% in the present study (57%) did not differ from the values previously reported by others, which ranged from 53.5%^4^ to 69%.^11^ It had been expected that parous women would perform better than nulliparae with regard to predicting birth weight, but the difference between them was insignificant in this study. This confirms the findings from other studies that evaluated the effect of parity on FWE.^3,4^ Several other factors, not evaluated in the present study, could potentially influence the accuracy of maternal FWE. However, other studies^1,11^ have previously reported that maternal characteristics such as age, education level, maternal weight and mean birth weight of children born previously did not affect the accuracy of maternal estimates. The effect of recent ultrasound scans on the mother's estimate should also be considered. Although this effect was not specifically evaluated in the present study, a previous paper^11^ did not find any correlation between the interval between the most recent antepartum ultrasound scan and the accuracy of maternal FWE among diabetic women.

One of the strong points of the present study is that it was the first to compare the accuracy of Dare's and Johnson's formulas with maternal FWE. It was also the first paper to compare the accuracy of these two clinical methods on the same set of patients and to compare the accuracy of Dare's formula with ultrasound FWE.

One potential bias in this study was that the patients were not consecutive and that only some of the patients delivered during the study period were enrolled. This could not be avoided, because of the rotation of the participating residents. A second potential limitation of the study was that four relatively inexperienced physicians performed all the clinical estimates, at various hours during their shifts in the labor ward. Ben-Aroya et al.^23^ reported that fatigue affected the accuracy of residents’ clinical FWE using abdominal palpation but, curiously, did not affect their ultrasound estimates This may suggest that more objective tasks, such as specific ultrasound measurements, are less influenced by fatigue than are subjective evaluations, such as abdominal palpation. If this is true, it is possible to speculate that clinical FWE using a specific technique involving measurements with a tape might also be less affected by fatigue. A third potential bias in the present study was that the ultrasound FWE was not performed by a single examiner. It was operationally impossible to have all ultrasound scans performed by a single individual. On the other hand, since the expertise of the ultrasound examiner may influence the accuracy of the FWE,^24^ the study was designed to ensure that all examinations were performed by the most qualified ultrasound specialist available at the time. One final limitation was that only Hadlock's formula was used for ultrasound FWE. Although some authors have advocated the use of other equations, a recent systematic review reported that the accuracy of Hadlock's formula did not differ significantly from other models.^25^ The fact that clinical and ultrasound estimates were obtained by different observers also precluded the possibility that one estimate could have influenced the other.

## CONCLUSION

The present study indicates that, among full-term singleton cephalic pregnancies, fetal weight estimation using a measuring tape and two different clinical formulas is just as accurate as maternal and ultrasound estimates for predicting the actual birth weight (± 10%). These simple clinical methods for FWE are easy to perform and teach and may be useful, inexpensive and practical tools for predicting birth weight, especially for less experienced examiners.
